# NanoMarine therapeutics: a new wave in drug delivery from oceanic bioresources targeting colon cancer via miRNA modulation

**DOI:** 10.3389/fgene.2025.1668618

**Published:** 2025-10-08

**Authors:** Ramkumar Muthu, Rajkumar Manickam, Rajkumar Thamarai, Sivabalan Sivasamy, Karthikeyan Mahendran, Rajkumar Prabhakaran

**Affiliations:** ^1^ Department of Biotechnology, Sri Kaliswari College (Autonomous), Sivakasi, Tamil Nadu, India; ^2^ Department of Biotechnology, Karpagam Academy of Higher Education (Deemed to be University), Coimbatore, Tamil Nadu, India; ^3^ Department of Respiratory Medicine, Saveetha Medical College and Hospital, Saveetha Institute of Medical and Technical Sciences (SIMATS), Saveetha University, Chennai, Tamil Nadu, India; ^4^ Department of Biochemistry, Karpagam Academy of Higher Education (Deemed to be University), Coimbatore, Tamil Nadu, India; ^5^ Department of Microbiology, PSG College of Arts & Science, Coimbatore, Tamil Nadu, India; ^6^ Centre for Cancer Research, Karpagam Academy of Higher Education, Coimbatore, Tamil Nadu, India

**Keywords:** colon cancer, nanomarine therapeutics, epigenetic regulation, targeted drug delivery, miRNA

## Abstract

The increasing global incidence of colorectal cancer (CRC) necessitates the development of innovative and targeted therapeutic interventions. Marine-derived bioactive compounds have gained prominence due to their structural diversity, intrinsic bioactivity, and potential to modulate oncogenic and tumor-suppressive microRNAs (miRNAs). Simultaneously, miRNAs have gained attention as critical regulators of gene expression in CRC, influencing key processes such as proliferation, metastasis, apoptosis, and chemoresistance. Nanotechnology has further transformed this field by enhancing drug solubility, stability, and tumor-specific delivery, thereby enabling combinatorial approaches such as the co-delivery of miRNA-targeted nano-formulations with conventional chemotherapeutics. Notably, co-delivery systems combining miRNA-targeted nano-marine drugs with conventional chemotherapy have shown synergistic effects in overcoming drug resistance and enhancing therapeutic efficacy. Despite encouraging preclinical outcomes, clinical translation remains constrained by challenges related to pharmacokinetics, scalability, immunogenicity, and regulatory compliance. This review critically evaluates the mechanistic interplay between marine compounds and miRNAs in CRC, advances in nanoformulation strategies, and translational barriers, providing insights into future directions for clinical application.

## Graphical Abstract

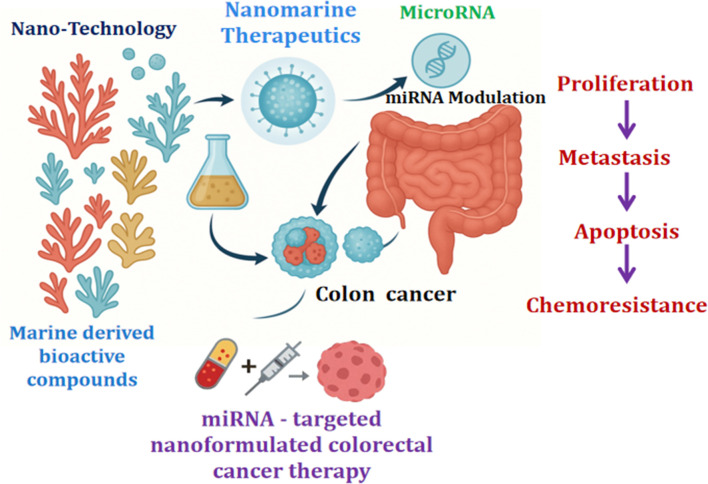

## Highlights

- CRC remains the most prevalent and deadly disease worldwide.
- Chemotherapy and targeted treatments remain constrained by toxicity, resistance, and the need for specific delivery.
- Significant advancement in therapy for CRC is alterations in miRNA levels.
- Marine-sourced bioactives are great because they absorb and work well for miRNA therapies.
- Marine nano-pharmaceuticals showed potential in treating life-threatening diseases.

## 1. Introduction

Nanotechnology-based drug delivery systems leverage nanoscale carriers, including liposomes, micelles, and polymeric nanoparticles, to encapsulate and convey therapeutic molecules to specific tissues. The integration of nanotechnology with marine-derived compounds has emerged as a transformative approach to provide innovative solutions to persistent challenges in therapeutic development (Sepe et al., 2025; Macha et al., 2019). Marine nano-pharmaceuticals, derived from natural marine products, have shown potential for the treatment of life-threatening diseases, including cancer, AIDS, and metabolic disorders. These cost-effective, biocompatible, and sustainable solutions underscore the importance of marine resources in advancing next-generation therapeutics (Macha et al., 2019). Marine biomaterials (polysaccharides, proteins, and lipids) exhibit distinct physicochemical and biological traits, making them ideal candidates for nanotechnology-based drug delivery platforms. These materials are abundant, biodegradable, and exhibit inherent therapeutic potential, which has spurred extensive research into their applications in nanomedicine.

Marine-derived nanomaterials, such as chitosan, alginate, and fucoidan, have been extensively studied for their ability to form stable nanostructures and facilitate controlled drug release. These systems not only improve drug efficacy but also minimize adverse effects by ensuring site-specific delivery (Thakur et al., 2024). Furthermore, advancements in microfluidic-based nanoparticle synthesis have enabled scalable production of these nanoparticles, paving the way for their clinical translation (Sepe et al., 2025). By elucidating the molecular mechanisms and pharmacokinetics of marine-basednano-drug delivery systems, researchers aim to accelerate their clinical translation and maximize their therapeutic potential (Wang et al., 2023). Recent advancements have demonstrated their efficacy in delivering drugs with enhanced precision, reduced toxicity, and improved bioavailability, particularly for complex diseases such as cancer, cardiovascular disorders, and neurological conditions (Yu et al., 2025).

Marine nanotechnology has significant potential, especially in the modulation of microRNAs. These small noncoding RNA molecules are crucial for regulating gene expression and are implicated in a variety of diseases. miRNAs-based therapeutics, including miRNA mimics and antisense inhibitors, have shown immense potential in targeting complex molecular pathways. However, challenges such as low stability, off-target effects, and toxicity at high doses have hindered their clinical adoption. Nanoformulated marine drugs offer a viable solution by providing safe, efficient, and targeted delivery of miRNA therapeutics. Recent research has highlighted the potential of marine-derived nanoparticles in encapsulating and delivering miRNA drugs with improved specificity and reduced side effects, representing a significant step forward in miRNA therapy (Kim and Croce, 2023; Brillante et al., 2024). The convergence of marine biotechnology and nanotechnology has also opened new avenues for addressing global health challenges. Among cancer types, CRC remains one of the most prevalent and deadly worldwide, with over 1.9 million new cases annually (Sung et al., 2021). Despite advances in chemotherapy and targeted treatments, therapeutic efficacy remains constrained by systemic toxicity, drug resistance, and suboptimal tumor-specific delivery. In this context, miRNA modulation has emerged as a novel genetic intervention strategy, with the potential to reshape aberrant gene networks involved in tumor proliferation, metastasis, and drug resistance (Zhang et al., 2022). Since miRNAs are master regulators of gene expression, their dysregulation profoundly alters cancer genetics by disrupting oncogenes, tumor suppressor genes, and signaling pathways central to CRC progression. CRC progression is strongly influenced by genetic alterations, including mutation in APC, KRAS, TP53 and SMAD4, as well as epigenetic changes that deregulate Wnt/β-catenin, MAPK, and TGF- β pathways. miRNAs intersect with these genetic networks by modulating oncogenes with tumor suppressors. Thus, integrating miRNA based modulation with marine nanotechnology provides a mechanistic framework to reprogram dysregulates genetic circuits in CRC.

Marine-derived nanomaterials (MDNMs) are unique due to their distinctive physicochemical properties, high biocompatibility, inherent bioactivity, and versatile targeting capabilities (Xin et al., 2025; Brar et al., 2021; Chai et al., 2025). MDNMs, which can be derived from chitosan, silica, and hybrid biopolymer systems, possess highly customizable size, shape, surface charge, and hydrophobicity. The ability of their nanoscale dimensions to penetrate biological barriers, transport across cell membranes, and accumulate in tumor tissues is facilitated by their nanoscale dimensions. Their unique elemental and molecular compositions, such as polysaccharides, peptides, and minerals, which are derived from marine sources, are often more responsive to pH, magnetic field, and Temperature. Surface modification is a standard method of improving stability, reducing aggregation, and targeting drug release in the tumor microenvironment (Yagublu et al., 2022; Deng et al., 2022; Alfareed et al., 2022). MDNMs are often tolerated due to their affinity for substances already present in the human body or diet, such as chitosan and alginate.

They are capable of being engineered to have a surface that is low toxicity, non-immunogenic, and selectively absorbed by cancer cells. Anticancer activity is inherent in specific nanomaterials that come from the marine environment, either through direct induction of apoptosis or by modulating tumor-related pathways. For instance, magnetoelectric nanocomposites with marine-derived protein layers exhibit both protective biocompatibility and growth inhibition in the face of CRC (Alfareed et al., 2022; Bandaru et al., 2025). The effectiveness of MDNMs in CRC therapy depends on both active and passive targeting. By using hyaluronic acid as a ligand, functionalization can take advantage of overexpressed receptors (CD44) on CRC cells, leading to selective binding and enhanced uptake. Marine-derived systems are designed to facilitate dual-response mechanisms synergistically; for instance, hyaluronidase-mediated deprivation or redox-sensitive drug release can ensure localization and regulated activation within the tumor microenvironment. Efficient targeting and delivery can be achieved through oral administration using bacterial bio-nanoparticle platforms, which are inspired by marine microbes (Xin et al., 2025; Chai et al., 2025; Brar et al., 2021).

However, clinical translation of miRNA-based therapy has been limited by challenges in achieving stable, targeted, and efficient delivery. Due to the challenges of achieving stable and targeted delivery, nanomaterials based on marine compounds offer a unique platform for miRNA therapy. Materials from algae, crustaceans, and marine sponges, particularly chitosan, alginate, fucoidan, and marine-based liposomes, are being explored as next-generation nano-biocarriers (Pagoni et al., 2023). Their ability to respond to tumor-specific stimuli (for example, pH, redox potential) and promote cellular uptake and intracellular release makes them particularly suitable for gastrointestinal cancers such as CRC. This review focuses on miRNA modulation as a pivotal therapeutic axis in CRC, exploring the intersection of marine biotechnology, nanomedicine, and cancer genetics. It critically evaluates recent advances in marine-derived nanoformulation designed for miRNA delivery, their mechanistic roles in modulating oncogenic or tumor-suppressor miRNAs, and the molecular rationale supporting their design. This is the first comprehensive review that expressly amalgamates marine-derived nanotechnology, modulation of microRNA (miRNA), and CRC therapy in a singular context. Although previous studies have investigated marine bioactives, the anticancer potential of marine bioactives, the role of miRNAs in CRC, or the application of nanocarriers individually, no review has previously engaged all three thematic areas in order to integrate them systematically. By converging these domains, our review points to an entirely new picture, helps elucidate the potential of marine-based nanoplatforms in targeting oncogenic and tumor-suppressor miRNAs, and addresses some translational issues and challenges for CRC control and management. This review establishes a new framework and provides an objective foundation for continued experimental and clinical investigations. Our review systematically integrates marine nanotechnology, miRNA modulation, and cancer genetics to highlight their convergence in CRC therapy.

## 2. miRNAs in CRC: roles, regulatory pathways, marine compound applications

### 2.1 General roles of miRNAs in CRC

**FIGURE 1:**
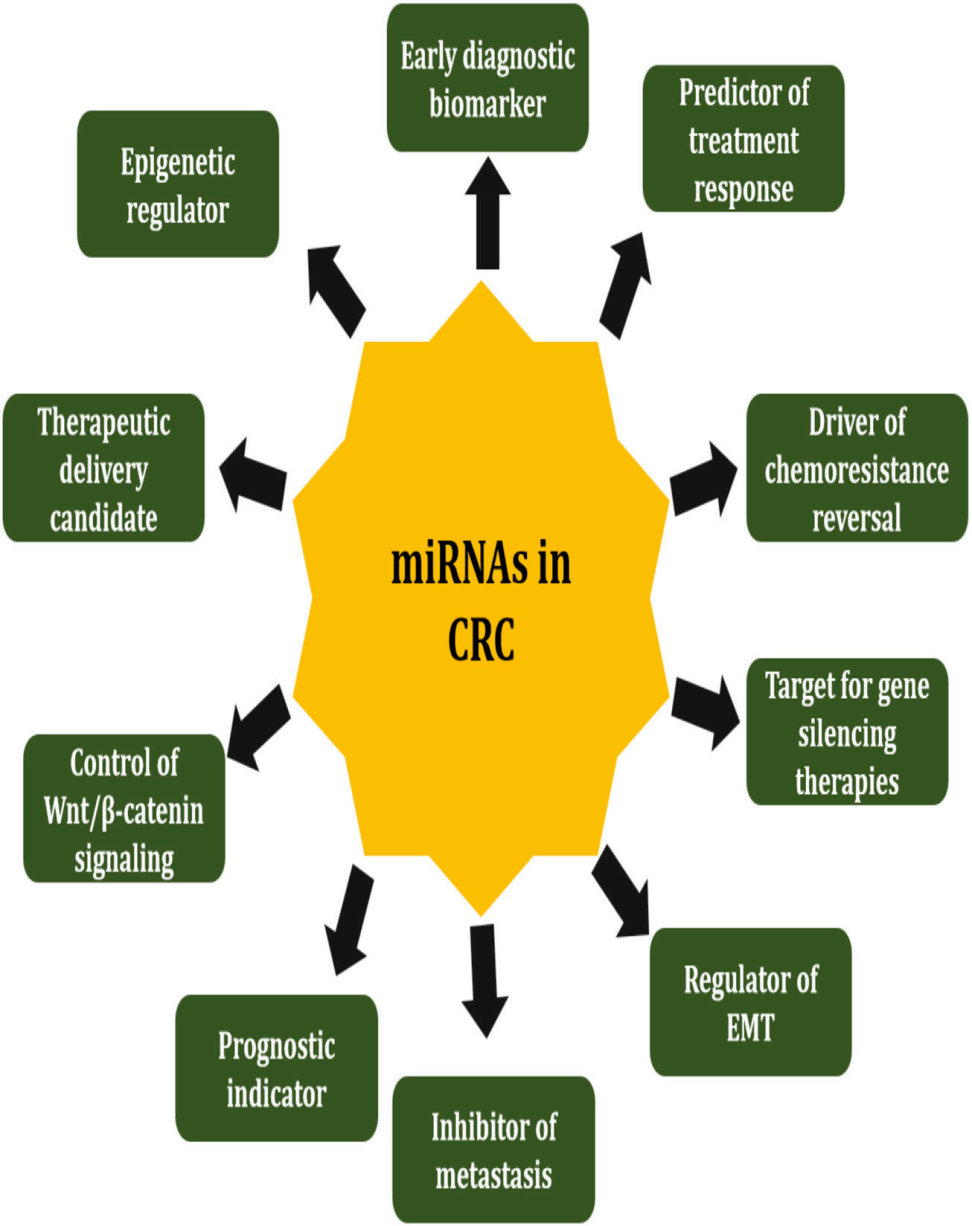
The various functions and application of miRNAs in CRC pathology and therapy. MiRNAs play a key regulaors of cancers by changing oncogenes and tumorsuppressors, influencing EMT, metastasis and chemoresistance. miRNAs also act as animportant biomarkers for diagnostic and prognostics. Additionally, miRNA can regulate immune responses, major signaling pathways like Wnt/beta catenin and also represent promising target or vehicles for gene therapy and epigenetic regulations.

CRC remains one of the most prevalent and lethal malignancies globally, with increasing incidence and mortality rates despite advancements in screening and therapeutic interventions. The pathogenesis of CRC has been influenced by miRNAs, which are small non-coding RNAs that are about 21–23 nucleotides in length and play a key role. Post-transcriptional modulation of gene expression by these molecules affects key cellular processes, including proliferation, apoptosis, angiogenesis, epithelial-to-mesenchymal transition (EMT), and chemoresistance. In CRC, their dysregulation has been extensively documented, and some miRNAs can act as either oncomiRs or tumor suppressor miRNAs (tsmiRs), depending on their role (Shirzad et al., 2025). miRNAs and oncogenic signaling pathways, such as the Wnt, Ras, and TGF-beta pathways, have a complex interplay, highlighting their critical role in CRC initiation, progression, and metastasis (Guo et al., 2017). Furthermore, Mehrgou et al. (2020) reported that miR-21, miR-143, and miR-200 have exhibited potential as diagnostic and prognostic biomarkers, providing insights into patients’ outcomes and therapeutic response. The translation of miRNA-based therapies into clinical practice is still challenging due to issues related to delivery, stability, and off-target effects, despite these promising findings (Figure 1).

### 2.2 Marine compound applications in CRC

The marine-derived compounds and nanotechnology have opened a novel avenue for miRNA modulation in CRC in recent times. The diverse bioactive properties of marine natural products, which have been demonstrated to have the ability to regulate miRNA expression, have exerted an anti-tumoral effect. For example, marine-derived drugs have been shown to exhibit changes in oxidative signaling and miRNA networks, offering potential therapeutic benefits while reducing the systemic side effects/toxicity in non-cancerous cells (Chuang et al., 2024). Furthermore, these marine nanoformulated drugs offer a promising platform for targeted miRNA delivery and enhanced therapeutic efficacy.

#### 2.2.1 Tumor suppressor miRNAs in CRC

Tumor suppressor miRNAs modulate the pathways related to cell growth, programmed cell death, and metastasis. Due to this process, tsmiRNA plays a significant role in inhibiting the progression of CRC. For example, miRNA-145 is routinely observed to be downregulated in CRC cases. Liu et al. (2023) report suggested that miRNA-145 is a key regulator of the mTOR signaling pathway, while inhibiting angiogenesis and tumor proliferation by targeting p70S6K1. Recently, researchers have identified that miR-148a targets BCL2, thereby facilitating apoptosis in CRC cells by targeting BCL2. Similarly, Liu et al. (2023) study demonstrated that let-7c has been shown to inhibit metastasis by acting on an enzyme involved in the remodeling of the extracellular matrix, MMP11. Furthermore, Ellakwa et al. (2024) reported that miR-22 is a major tumor suppressor that is responsible for modulating the TGF/SMAD signaling pathway, which is crucial in maintaining cellular homeostasis and enhancing responsiveness to immunotherapy in CRC. These findings demonstrate the crucial significance of focusing on tumor suppressor pathways in combating the progression of CRC.

### 2.3 Oncogenic miRNAs (oncomiRs) in CRC

Overexpression of oncomiRs, also known as oncogenic microRNAs, occurs in CRC and plays a crucial role in tumor development by targeting tumor suppressor genes. miR-21, a well-studied oncomiR, is a prime example of how it accelerates CRC progression by inhibiting PTEN, a vital tumor suppressor that regulates the PI3K/AKT signaling pathway (Doghish et al., 2025). Similarly, Liu et al. (2023) demonstrated that miR-17, another oncomiR, affects tumor growth by targeting RND3 (a regulator of cellular proliferation). Ellakwa et al. (2024) reported that miR-142-3p activates the RAC1-ERK1/2 signaling pathway, which facilitates EMT and metastasis in CRC. These findings demonstrate that microRNAs play a complex role in CRC and that their dysregulation can either inhibit or enhance tumor growth, based on their specific targets and expression levels. EMT plays a vital role in CRC metastasis, and miRNAs play a significant role in its regulation. Park et al. (2008) reported that the miR-200c (miR-200 family member) has been identified as an inhibitor of EMT by targeting transcription factors such as ZEB1 and ZEB2 that suppress E-cadherin expression. A decrease in miR-200 levels has been linked to an increase in invasiveness and a lower prognosis for CRC patients. Conversely, oncogenic miRNAs promote EMT by inhibiting tumor suppressors like PDCD4 and TIMP3, respectively (Doghish et al., 2025).

### 2.4 miRNAs in EMT and metastasis

The degradation of the extracellular matrix and cell motility are made easier by these miRNAs, which in turn promote the spread of cancer cells. The dynamic interaction between tumor suppressive and oncogenic miRNAs in regulating EMT highlights their potential as therapeutic targets to prevent metastasis. The gut microbiome has been found to play a significant role in regulating miRNA expression, which could impact the development and progression of CRC, according to recent research (Shirzad et al., 2025). Recently, Shirzad et al. (2025) reported that microbial imbalances, or dysbiosis, can cause oncomiRs, such as miR-21, to rise, while at the same time lowering the levels of tumor suppressor miRNAs, like miR-145, thus creating an environment that is favorable for the growth of tumors. In addition, microbial components and inflammatory cytokines activate miR-155, a significant miRNA, which may be involved in chronic inflammation and induce the progression of CRC. On a positive note, some probiotics have exhibited promise in restoring the expression of tsmiRNAs, opening the way to a significant microbiome-based treatment option for CRC. Recently, Li et al. (2025) demonstrated that probiotics can control the expression of host miRNAs, which in turn affects important immune pathways and helps to maintain gut integrity and lower inflammation. Specifically, it has been explained that therapeutic probiotics exert their beneficial effects through miRNA-mediated mechanisms that control gene expression involved in immune responses, the function of the epithelial barrier, and microbial interactions (Davoodvandi et al., 2021).

### 2.5 miRNA clusters in CRC

On the other hand, the regulation of pathways associated with CRC is facilitated by microRNA clusters, which consist of multiple miRNAs derived from a single transcript. The miR-130a/301a/454 cluster is a noteworthy instance, as it targets SMAD4, a crucial regulator of the TGF beta signaling pathway, and thus facilitates the progression of CRC (Liu et al., 2023). Likewise, in CRC, the miR-17–92 cluster is associated with key functions such as angiogenesis, cell proliferation, and immune evasion. Al-Nakhle (2024) emphasized this cluster’s potential as a biomarker candidate and mechanistic participant, highlighting its complex involvement in CRC. Likewise, MohajeriKhorasani et al. (2024) demonstrated that the miR-17-92a-1 cluster host gene function is a crucial regulator in CRC genesis and progression, underscoring its clinical significance. According to Meng et al. (2015), synchronous CRCs have differential expression of the miR-17-92 and miR-143–145 clusters, indicating their role in tumor heterogeneity and disease dynamics. Furthermore, Ellakwa et al. (2024) reported that various microRNAs, including members of the miR-17–92 cluster, play a critical role in essential cancer-related processes such as cell proliferation, angiogenesis, apoptosis, and chemoresistance, highlighting the complex regulatory network in CRC pathophysiology.

## 3 Marine bioactives as emerging cancer therapeutics

Marine-derived bioactives have shown promise for the development of novel anti-cancer treatments due to their unique structures and ability to work differently from standard chemotherapy (Conte et al., 2020). Many of these compounds exhibit potent cytotoxic, anti-proliferative, anti-angiogenic, and apoptosis-inducing effects against a wide range of cancers, including colon, breast, lung, and hematological malignancies. In recent decades, there have been significant enhancements in the isolation and characterization of marine natural products (MNPs) (Jiménez, 2018). For example, Trabectedin (Yondelis^®^), which comes from the sea creature *Ecteinascidiaturbinata*, along with Brentuximabvedotin, which is an antibody-drug conjugate that incorporates the marine toxin dolastin 10, are FDA-approved medications that underscore the clinical importance of these bioactives from marine (Malve, 2016). In addition, Reed et al. (2007) reported that the marine actinomycete*Salinisporatropica* synthesized potent proteasome inhibitor *Salinosporamide* A, which has exhibited promising results in clinical trials for multiple myeloma. Furthermore, Leisch et al. (2018) reported that didemnin B, sourced from the tunicate *Trididemnumsolidum*, and aplidine (plitidine), isolated from *Aplidiumalbicans*, exert notable antitumor activity by inducing cell cycle arrest, inhibiting eEF1A2, and triggering mitochondrial-dependent apoptosis.

**FIGURE 2:**
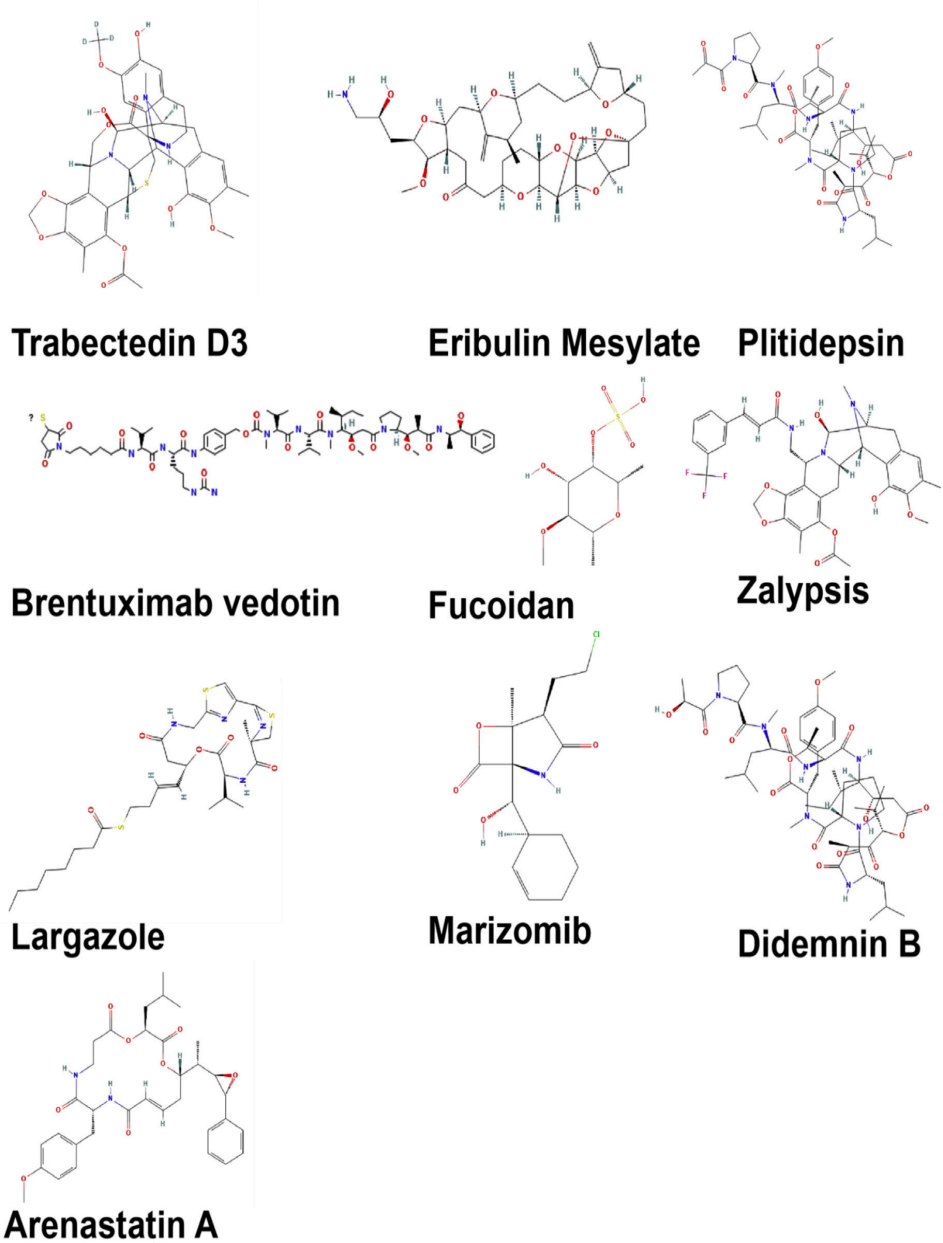
Marine’s compounds secondary structures obtained from PubChem Database.

A variety of structurally distinct compounds, including halichondrin B, manzamine A, and bryostatin 1, are being produced by marine microalgae, cyanobacteria, sponges, and mollusks (Figure 2). According to Wu et al. (2021), these compounds are capable of influencing key cancer-associated signaling pathways, including PI3K/Akt/mTOR, NF-kappaB, and MAPK, thereby presenting a multi-targeted therapeutic approach that may address the drawbacks of conventional single-target chemotherapy. Marine-derived bioactives, specifically peptides and polysaccharides, including fucoidans from brown algae as well as laminarins, have been reported to have immunomodulatory effects that help and enhance cancer immunotherapy by effectively aligning it with modern approaches to precision and personalized medicine. Recent advancements in marine biotechnology, deep-sea metagenomics, and synthetic biology have significantly improved the ability to sustainably extract and manipulate these natural products, facilitating scalable production with reduced environmental impact (Jiménez, 2018). The marine-derived bioactives utilized in cancer therapy are presented in Table 1.

**TABLE 1:** Marine-derived bioactive compounds in cancer therapy.

Compound	Source organism	Chemical class	Mechanism of action	Targeted cancers	References
Trabectedin (ET-743)	*Ecteinascidiaturbinata* (sea squirt)	Tetrahydroisoquinoline alkaloid	Binds to DNA minor groove, disrupts transcription and DNA repair	Soft tissue sarcoma, ovarian cancer	D'Incalci and Galmarini (2010)
Eribulinmesylate	Synthetic analog of halichondrin B from *Halichondriaokadai*	Macrocyclic ketone	Inhibits microtubule dynamics, leading to G2/M cell cycle arrest	Metastatic breast cancer, liposarcoma	Towle et al. (2001)
Plitidepsin (Aplidin)	*Aplidiumalbicans* (tunicate)	Cyclic depsipeptide	Inhibits eEF1A2, leading to oxidative stress and apoptosis	Multiple myeloma	Alonso-Álvarez et al. (2017)
Brentuximabvedotin	Marine cyanobacteria derivative (MMAE)	Antibody-drug conjugate	Anti-CD30 mAb linked to MMAE, disrupts tubulin polymerization	Hodgkin’s lymphoma, anaplastic large cell lymphoma	Younes et al. (2010)
Zalypsis	*Jorunnafunebris* (sea hare)	Alkaloid	DNA minor groove binder, induces DNA double-strand breaks	Sarcoma, myeloma, carcinoma	Ocio et al. (2009)
Largazole	Symploca sp. (marine cyanobacteria)	Thioester-containing depsipeptide	HDAC inhibitor, induces cell cycle arrest and apoptosis	Colon, pancreatic, and breast cancer	Liu et al. (2010)
Marizomib (Salinosporamide A)	*Salinisporatropica* (marine actinomycete)	β-lactone-γ-lactam	Irreversible 20S proteasome inhibitor	Glioblastoma, multiple myeloma	Macherla et al. (2005)
Didemnin B	*Trididemnumsolidum* (tunicate)	Cyclic depsipeptide	Inhibits protein synthesis by targeting EF1A	Leukemia, solid tumors	Newman and Cragg (2004)
Fucoidan	Brown seaweeds (*Fucusvesiculosus*)	Sulfated polysaccharide	Anti-angiogenic, immunomodulatory, apoptosis inducer	Breast, colon, and liver cancers	Reyes et al. (2020)
Arenastatin A	*Dysideaarenaria* (sponge)	Peptide	Microtubule destabilizer	Solid tumors	Mulder et al. (2013)

Marine-derived bioactive compounds have also demonstrated significant potential as modulators of miRNA expression in CRC. For example, chlorogenic acid, sourced from marine organisms, has been shown to decrease the levels of miR-21, which subsequently inhibits the progression of early-stage CRC in preclinical studies (Doghish et al., 2025). Furthermore, other marine-derived substances, such as fucoidan and astaxanthin, have also been found to affect miRNA expression and impede CRC-related processes, particularly inflammation and angiogenesis. These compounds present new opportunities for the development of natural miRNA-based therapies for CRC. By leveraging the unique properties of marine-derived compounds, researchers can explore innovative strategies for modulating miRNA, potentially enhancing both preventive and therapeutic approaches against CRC.

### 3.1 Marine-derived compounds targetingmiRNA-driven oxidative stress in CRC

Nowadays, the bioactive compounds from marine compounds are recognized as effective modulators of oxidative stress through the regulation of miRNAs in CRC. For instance, certain marine drugs can induce oxidative stress by down-regulating antioxidant miRNAs such as miR-210 and miR-155, which are often over-expressed in CRC and contribute to tumor survival. These miRNAs play a crucial role in regulating essential antioxidant enzymes, including superoxide dismutase (SOD) and catalase, which are vital for maintaining redox balance in cancer cells. Specific marine compounds, such as fucoxanthin and sargachromenol, have been shown to influence these miRNAs, leading to increased oxidative stress and the induction of apoptosis in cancer cells (Chuang et al., 2024). Furthermore, bioinformatics tools like miRDB have been employed to identify additional miRNA targets of these compounds, thereby enhancing their therapeutic potential. Chuang et al. (2024) explored various marine anticancer agents capable of targeting redox-sensitive signaling through the modulation of miRNA, thereby improving antioxidant responses as well as reducing tumor growth. This dual mechanism directs antioxidant signaling and miRNA-based regulation, positioning marine compounds as promising candidates for integrative cancer therapeutics. Notably, Basak et al. (2020) emphasized oxidative stress as a central driver in CRC pathogenesis, supporting the rationale for antioxidant-based intervention. *Spirulina platensis* filtrates, as reported by Smieszek et al. (2017), modulate apoptosis-associated miRNAs and mRNAs in CRC cell lines, offering further evidence of marine bioactives’ regulatory potential. Moreover, emerging studies, such as that by Ngum et al. (2023), have systematically reviewed natural compounds capable of altering non-coding RNA expression in inflammation and oxidative stress-related disorders, reinforcing the broader relevance of miRNA modulation in disease mitigation. Dyshlovoy. (2021) further identified recent marine-derived candidates with cancer-preventive potential, underscoring a growing pharmacopeia of marine bioactives for oxidative stress-targeted therapy. Together, these findings affirm the role of marine-derived molecules not only as antioxidants but also as sophisticated regulators of miRNA networks relevant to CRC progression and resistance.

### 3.2 Marine compounds modulating miRNA clusters in CRC

In contrast to the modulation of individual miRNAs, marine compounds have been identified as agents capable of targeting miRNA clusters, collections of co-expressed miRNAs that exert synergistic effects on cancer pathways. One notable example is the miR-17–92 cluster, often referred to as an “oncomiR cluster”, which is overexpressed in CRC and promotes cell proliferation and metastasis. Marine-derived substances, such as fucoidan, have been shown to downregulate this cluster, effectively inhibiting multiple oncogenic pathways simultaneously (Doghish et al., 2025). Additionally, marine compounds also influence miRNA clusters involved in EMT, such as the miR-200 family. For instance, fucoxanthin has been shown to upregulate the miR-200 family, counteracting EMT and reducing the metastatic potential of CRC cells. This multifaceted targeting underscores the unique therapeutic potential of marine compounds in miRNA-based treatments for CRC. Table 2 provides a summary of the expression patterns of specific miRNAs that are dysregulated in CRC, categorized as either upregulated or downregulated. The table includes details on their respective miRNA cluster memberships, biological functions, and the sequences of forward and universal reverse primers (5′-3′) used for quantitative PCR detection. Proprietary assay information is included where applicable. Primary sources consist of OriGene, Invitrogen/Thermo Fisher, and various published literature databases, including PubMed, Oncotarget, and Nature. This table serves as a valuable reference for researchers seeking to validate miRNA expression in CRC using qPCR techniques.

**TABLE 2:** Overview of upregulated and downregulated microRNAs (miRNAs) in CRC with cluster association, functional role, and primer details for RT-qPCR.

miRNA	Regulation	Cluster/notes	Function	Forward primer (5′→3′)	Reverse primer (universal)	Source and notes
miR-17-5p	Up	miR-17/92a-1	Oncogenic; promotes proliferation, inhibits apoptosis, angiogenesis regulator	TGCTTACAGTGCAGGTAG	GAA​CAT​GTC​TGC​GTA​TCT​C	Origene human qSTAR kit
miR-18a-5p	Up	miR-17/92a-1	Enhances tumor progression; linked to metastasis and immune evasion	GAT​AGC​AGC​ACA​GAA​ATA​TTG​GC	GTGCAGGGTCCGAGGT	Commercial RT-qPCR assay
miR-19a-3p	Up	miR-17/92a-1	Key oncogene; targets PTEN, promotes cell survival	GTT​TTG​CAT​AGT​TGC​ACT​A	GAA​CAT​GTC​TGC​GTA​TCT​C	Origene human kit
miR-20a-5p	Up	miR-17/92a-1	Regulates E2F1, promotes proliferation, cell cycle progression	GCC​CGC​TAA​AGT​GCT​TAT​AGT​G	GTGCAGGGTCCGAGGT	Commercial assay
miR-19b-1-5p	Up	miR-17/92a-1	Promotes EMT and metastasis; PI3K/AKT signaling	TGT​TGC​ATG​GAT​TTG​CAC​A	GTGCAGGGTCCGAGGT	Commercial assay
miR-92a-1-5p	Up	miR-17/92a-1	Oncogenic; involved in angiogenesis and Wnt/beta catenin signaling	GTG​GTA​GGT​TGG​GAT​CGG​T	GTGCAGGGTCCGAGGT	Commercial assay
miR-106a-5p	Up	miR-106a/363	Oncogenic; suppresses tumor suppressor genes like p21	Proprietary AAAAGTGCTTACAGTGCAGGTAG	GTGCAGGGTCCGAGGT	Commercial assay
miR-18b-5p	Up	miR-106a/363	Facilitates proliferation; enhances metastatic potential	Proprietary TAAGGTGCATCTAGTGCAGATAG	GTGCAGGGTCCGAGGT	Commercial assay
miR-20b-5p	Up	miR-106a/363	Modulates Hypoxia-inducible factor 1 alpha (HIF-1alpha) and VEGF; promotes hypoxia adaptation	Proprietary CAAAGTGCTCATAGTGCAGGTAG	GTGCAGGGTCCGAGGT	Commercial assay
miR-19b-2-5p	Up	miR-106a/363	Similar to miR-19b-1; promotes cell survival via PTEN targeting	Proprietary TGTGCAAATCCATGCAAAACTGA	GTGCAGGGTCCGAGGT	Commercial assay
miR-92a-2-5p	Up	miR-106a/363 cluster	Enhances cell growth; Wntsignaling activation	AGG​TGG​GGA​TTA​GTG​CCA​TTA	GAA​CAT​GTC​TGC​GTA​TCT​C	OriGene rat kit
miR-363-5p	Up	miR-106a/363 cluster	Dual role; context-dependent, often upregulated in CRC and promotes proliferation	GGTGGATCACGATGCAA	GAA​CAT​GTC​TGC​GTA​TCT​C	OriGene human kit
miR-106b-5p	Up	miR-106b/93/25 cluster	Oncogenic; targets RB1 and p21, linked to therapy resistance	AAA​GTG​CTG​ACA​GTG​CAG​A	GAA​CAT​GTC​TGC​GTA​TCT​C	OriGene mouse
miR-93-5p	Up	miR-106b/93/25 cluster	Promotes angiogenesis, EMT, and cell cycle progression	CAAAGTGCTGTTCGTGC	GAA​CAT​GTC​TGC​GTA​TCT​C	OriGene human Or: AGG​CCC​AAA​GTG​CTG​TTC​GT/GTG​CAG​GGT​CCG​AGG​T
miR-25-3p	Up	miR-106b/93/25 cluster	Regulates apoptosis, enhances invasion and metastasis	CGGAGACTTGGGCAATT	GAA​CAT​GTC​TGC​GTA​TCT​C	OriGene human kit
miR-181a-5p	Up	miR-181 cluster	Modulates Wnt pathway; implicated in CRC progression	GCG​GTA​ACA​TTC​AAC​GCT​GTC​G	GTGCAGGGTCCGAGGT	Invitrogen/Thermo primer
miR-181b-5p	Up	miR-181 cluster	Suppresses tumor suppressors like CYLD; enhances NF-kappa B signaling	TTCATTGCTGTCGGTGG	GAA​CAT​GTC​TGC​GTA​TCT​C	OriGene human kit Or: GCG​GAT​CAT​TCA​TTG​CTG​TCG/ATC​TGG​TGG​CTC​TCG​GAG​TAA
miR-183-5p	Up	miR-183/96/182 cluster	Promotes migration, invasion, and anti-apoptotic activity	ATGGCACTGGTAGAATTC	GAA​CAT​GTC​TGC​GTA​TCT​C	OriGene human kit Or (rat): TAT​GGC​ACT​GGT​AGA​ATT​CAC
miR-96-5p	Up	miR-183/96/182 cluster	Targets FOXO1; oncogenic and chemoresistance mediator	Proprietary TTT​GGC​ACT​AGC​ACA​TTT​T	GAA​CAT​GTC​TGC​GTA​TCT​C	OriGene
miR-182-5p	Up	miR-183/96/182 cluster	Regulates metastasis, DNA repair; supports tumor growth	Proprietary GGCAATGGTAGAACTCAC	GAA​CAT​GTC​TGC​GTA​TCT​C	OriGene
miR-203a-3p	Up	miR-203a/203b cluster	Tumor suppressor; CRC proliferation and migration	CGGCGTGAAATGTTTAGG	GTGCAGGGTCCGAGGT	qPCR primer from mouse study
miR-203b-5p	Up	miR-203a/203b cluster	Similar function to miR-203a	TAG​TGG​TCC​TAA​ACA​TTT​CAC	GAA​CAT​GTC​TGC​GTA​TCT​C	OriGene primers
miR-222-3p	Up	miR-222/221 cluster	Metastasis, invasion	CTCAGTAGCCAGTGTAG	GAA​CAT​GTC​TGC​GTA​TCT​C	OriGeneqSTAR kit
miR-221-3p	Up	miR-222/221 cluster	Oncogenic, cell migration	ACCTGGCATACAATGTAG (OriGene) OR GGG​AAG​CTA​CAT​TGT​CTG​C (Takara bio)	GAA​CAT​GTC​TGC​GTA​TCT​C (OriGene) OR CAGTGCGTGTCGTGGAGT (Takara)	OriGene
miR-21-5p	Up		Oncogenic, therapy resistance	GCT​TAT​CAG​ACT​GAT​GTT​G OR ACG​TGT​TAG​CTT​ATC​AGA​CTG	GAA​CAT​GTC​TGC​GTA​TCT​C	OriGene
miR-31-5p	Up		Oncogenic in CRC	GCAAGATGCTGGCATAG	GAA​CAT​GTC​TGC​GTA​TCT​C	OriGene
miR-191-5p	Up	miR-191/425 cluster	Biomarker, regulatory functions	CGGAATCCCAAAAGCAG	TGT​CGT​GGA​GTC​GGC​AAT​TG	OriGene
miR-425-5p	Up	miR-191/425 cluster	Proliferation, metabolism	ATGACACGATCACTCCC	GAA​CAT​GTC​TGC​GTA​TCT​C	OriGene kit
miR-23a-3p	Up	miR-23a/27a/24–2 cluster	Oncogenic	TTCCTGGGGATGGGATT	GAA​CAT​GTC​TGC​GTA​TCT​C	OriGene kit
miR-27a-3p	Up	miR-23a/27a/24–2 cluster	Oncogenic	GGCTTAGCTGCTTGTGA	GAA​CAT​GTC​TGC​GTA​TCT​C	OriGene kit
miR-24-2-3p	Up	miR-23a/27a/24–2 cluster	Oncogenic	GCCTACTGAGCTGATATC	GAA​CAT​GTC​TGC​GTA​TCT​C	OriGene kit
miR-29b-1	Up	miR-29b-1/29a cluster	Tumor suppressor; ECM, metastasis	CTTCAGGAAGCTGGTTTC	CTCCTAAAACACTGATTT	Human qPCR primers
miR-29a	Up	miR-29b-1/29a cluster	Tumor suppressor; ECM, metastasis	CTG​ATT​TCT​TTT​GGT​GTT​C	GAA​CAT​GTC​TGC​GTA​TCT​C	Human qPCR primers
miR-301b	Up	miR-301b/130b cluster	Oncogenic	GTG​CAA​TGA​TAT​TGT​CAA​AG	GAA​CAT​GTC​TGC​GTA​TCT​C	OriGene kit
miR-130b	Up	miR-301b–130b cluster	Oncogenic	CTCTTTCCCTGTTGCAC	GAA​CAT​GTC​TGC​GTA​TCT​C	OriGene kit
miR-452	Up	miR-452/224 cluster	Oncogenic/metastasis	ACTGTTTGCAGAGGAAAC	GAA​CAT​GTC​TGC​GTA​TCT​C	OriGene kit
miR-224	Up	miR-452/224 cluster	Oncogenic/metastasis	AAG​TCA​CTA​GTG​GTT​CCG​T	GAA​CAT​GTC​TGC​GTA​TCT​C	OriGene kit
miR-145-5p	Down	Tumor suppressor	Inhibits proliferation, metastasis	GTCCAGTTTTCCCAGGA	GAA​CAT​GTC​TGC​GTA​TCT​C	Origene human primers; RT primer
miR-195-5p	Down	Tumor suppressor	Inhibits proliferation (CRC, others)	GAA​TTC​GCC​TCA​AGA​GAA​CAA​AGT​GGA​G	AGA​TCT​CCC​ATG​GGG​GCT​CAG​CCC​CT	Primer sequences published
miR-139-5p	Down		Tumor suppressor	AGTGCACGTGTCTCCAG	GAA​CAT​GTC​TGC​GTA​TCT​C	OriGene kit
miR-378a-5p	Down		Tumor suppressor/metabolism	CCTGACTCCAGGTCCT	GAA​CAT​GTC​TGC​GTA​TCT​C	OriGene kit
miR-143-3p	Down		Tumor suppressor	GCAGTGCTGCATCTCTG	GAA​CAT​GTC​TGC​GTA​TCT​C	OriGene kit

### 3.3 Therapeutic implications and challenges of miRNA-based treatments for CRC and miRNA modulation in chemoresistancetherapeutic target

**FIGURE 3:**
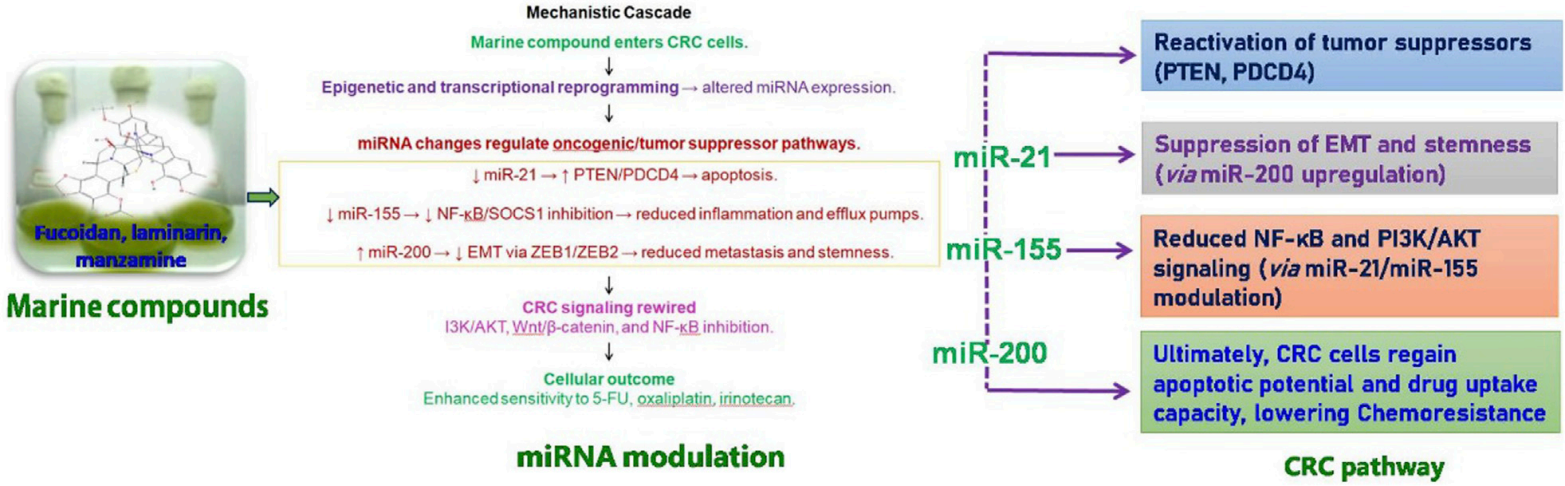
Mechanistic role of marine compounds in miRNA modulation and colorectal cancer (CRC) pathway regulation. Marine-derived compounds such as fucoidan, laminarin, and manzamine enter CRC cells and induce epigenetic and transcriptional reprogramming, leading to altered miRNA expression. These changes regulate key oncogenic and tumor suppressor pathways: upregulation of miR-21 inhibits PTEN/PDCD4 and promotes apoptosis; modulation of miR-155 reduces inflammation (via NF-κB/SOCS1 inhibition) and efflux pump activity; while miR-200 suppresses EMT and ZEB1/ZEB2, reducing metastasis and stemness. Collectively, these miRNA-mediated effects contribute to CRC signaling rewiring, including reduced NF-κB and PI3K/AKT activity, enhanced sensitivity to chemotherapeutics (e.g., 5-FU, cisplatin, irinotecan), and reactivation of tumor suppressors. Ultimately, CRC cells regain apoptotic potential and decreased drug resistance, lowering chemoresistance and improving therapeutic outcomes.

CRC treatment often faces considerable obstacles due to chemoresistance, which is affected by irregular miRNA expression. Key miRNAs, including miR-21, miR-155, and the miR-200 family, are crucial in influencing the mechanisms of chemo resistance (Figure 3) (Valenzuela et al., 2025). Targeting these miRNAs has the potential to mitigate chemoresistance. Notably, miR-200c, which functions as a tumor-suppressor miRNA, has been demonstrated to reverse EMT and enhance CRC cell sensitivity to chemotherapy by downregulating ZEB1 and ZEB2 (Valenzuela et al., 2025). Although therapeutic approaches utilizing miRNA mimics or inhibitors present a viable option for restoring chemosensitivity, challenges such as off-target effects and efficient delivery continue to pose significant hurdles. The summary of marine-derived compounds and their targeted miRNAs was listed in Table 3.

**TABLE 3:** Summary of Marine-derived compounds and their targeted miRNAs.

S. No	Compound/agent	Marine source	Targeted miRNA(s)	Regulation	Reported biological effect/model	References
1	Fucoidan	Brown seaweed (*Laminaria*/*Sargassum*)	miR-29b	Up	Anti-metastatic/anti-TGF-β in HCC cells (DNMT3B–MTSS1 axis)	Yan et al. (2015)
2	Fucoidan (clinical, oral)	Brown seaweed extract	Multiple circulating miRNAs	Mixed	Immune modulation and QoL improvement in cancer patients; altered plasma miRNA profile	Gueven et al. (2020)
3	Astaxanthin	Microalgae/krill carotenoid	miR-29a-3p	Up	Antifibrotic/antihypertrophic signaling in heart	Li et al. (2023)
4	Astaxanthin	Microalgae/krill carotenoid	miR-31-5p	Up	Anti-melanoma migration/invasion effects (cell model)	Gil-Zamorano et al. (2014)
5	Eicosapentaenoic acid (EPA)	Marine omega-3 (fish/algae)	miR-19b	Down	Anti-inflammatory reprogramming of human macrophages	Ramalho et al. (2020)
6	Eicosapentaenoic acid (EPA)	Marine omega-3 (fish/algae)	let-7a-5p	Up	Anti-proliferative signaling in endometrial cancer cells	Recchiuti et al. (2011)
7	Docosahexaenoic acid (DHA)	Marine omega-3 (fish/algae)	miR-21; miR-155 (and others)	Modulates	Anti-inflammatory/anti-tumor pathways; broad miRNA regulation reported	Mandal et al., 2012; Gutiérrez et al., 2019
8	Docosahexaenoic acid (DHA)	Marine omega-3 (fish/algae)	miR-155	Down	Inhibits VSMC migration and proliferation	Yin et al. (2020)
9	Resolvin D1 (RvD1)	DHA-derived SPM	miR-146b-5p	Up	Pro-resolving, anti-inflammatory macrophage phenotype	Zhang et al. (2021)
10	Resolvin D1 (RvD1)	DHA-derived SPM	miR-219-5p	Down	Resolution of acute inflammation in human macrophages	Fredman et al. (2012)
11	Maresin 1 (MaR1)	DHA-derived SPM	miR-21; miR-181b	Up	Anti-inflammatory, endothelial protection	Xi et al. (2018)
12	Protectin D1	DHA-derived SPM	miR-210	Up	Cardioprotection via HIF/mitochondrial pathways	Bei et al. (2024)
13	Resolvin E1 (RvE1)	EPA-derived SPM	miR-219-5p	Down	Pro-resolution signaling in macrophages	Fredman et al. (2012)
14	Eribulin (Halichondrin B derivative)	Marine sponge–derived anticancer drug	miR-195	Up	Anti-proliferative/chemosensitizing in breast cancer	OKAZAKI et al. (2018)
15	Dieckol (phlorotannin)	Brown alga Ecklonia cava	miR-134	Up	Antifibrotic effects (hepatic stellate cells)	Lee et al. (2016)
16	Alginate oligosaccharide	Brown seaweed cell wall	miR-29b	Modulates	Anti-inflammatory effects post–endovascular aneurysm repair (EVAR)	Yang et al. (2017)
17	Chitosan oligosaccharide	Crustacean/fungal chitin derivative	miR-132-5p	Up	Promotes peripheral nerve regeneration (schwann cell model)	Zhao et al. (2023)
18	Sargassumfusiforme polysaccharides	Brown seaweed	miR-92a-3p	Up	Enhances intestinal Muc2 via Notch1–Hes1 axis; protects against infection	Qin et al. (2025)
19	Sargassumfusiforme polysaccharides	Brown seaweed	miR-92a-3p→Notch1	Targets	Microbiota-driven barrier homeostasis	Qin et al. (2025)
20	Ulvan (Ulva lactuca sulfated polysaccharide)	Green seaweed	miR-542-3p	Up	Suppresses hepatocellular carcinoma growth (*in vitro*/*in vivo*)	Qiu et al. (2025)
21	Ulvan (Ulva lactuca)	Green seaweed	miR-542-3p→SLC35F6	Targets	Inhibits HCC proliferation and invasion	Qiu et al. (2025)
22	C-Phycocyanin	Marine/freshwater cyanobacteria (*spirulina*)	miR-642a-5p	Up	Inhibits NSCLC via RIPK1/NF-κB axis	Hao et al. (2021)
23	C-Phycocyanin	Spirulina	miR-3150a-3p	Up	Suppresses NSCLC via TIRAP axis (miRNA-seq)	Hao et al. (2022)
24	C-Phycocyanin	Spirulina	miR-627-5p	Up	Contributes to anti-NSCLC activity (miRNA-seq)	Hao et al. (2022)
25	C-Phycocyanin	Spirulina	miR-6883-3p	Up	Contributes to anti-NSCLC activity (miRNA-seq)	Hao et al. (2022)

### 3.4 Nanotechnology in miRNA-based therapeutics

**FIGURE 4:**
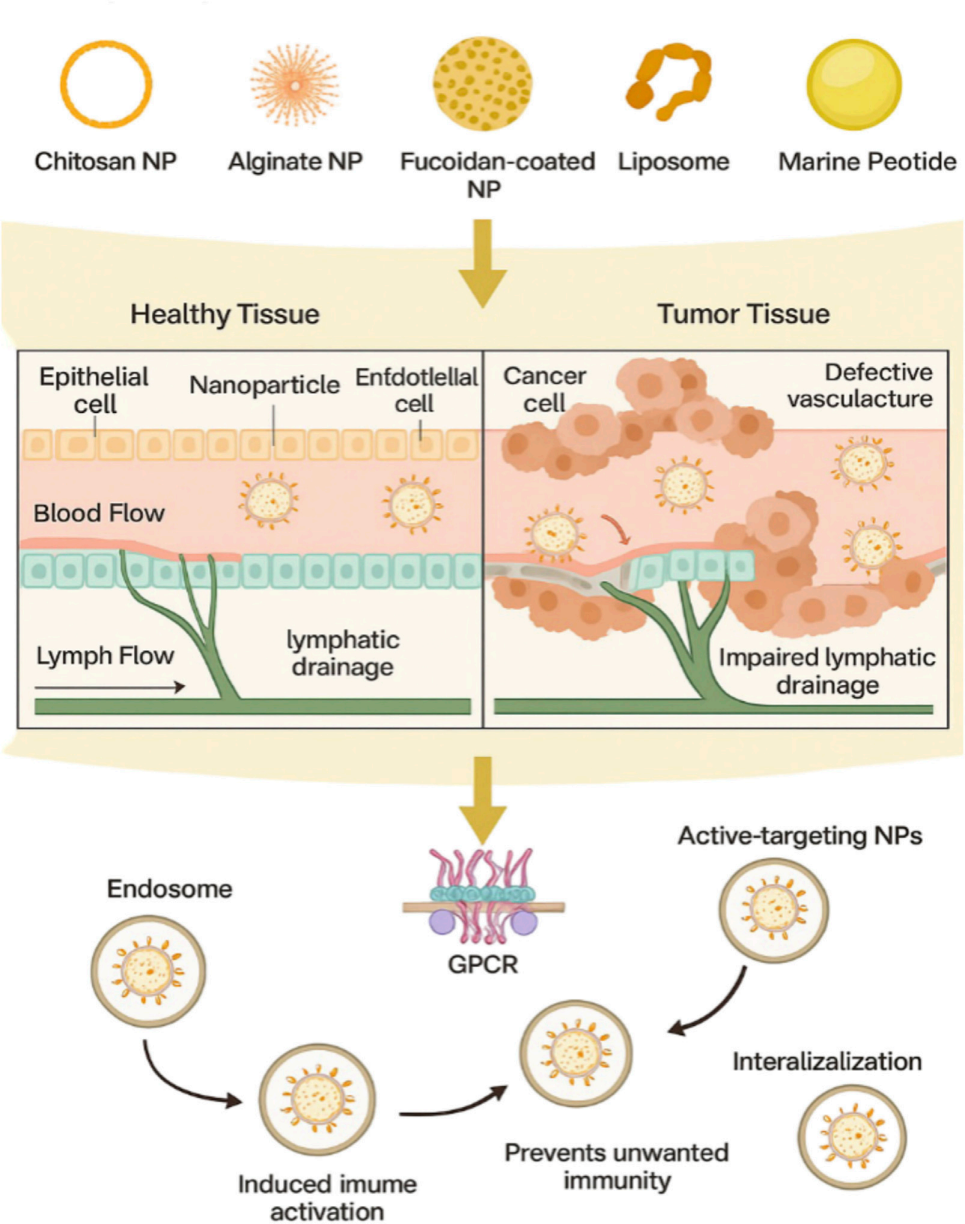
Schematic representation for targeted delivery of marine derived nanoparticles to tumor tissue and their cellular internalization mechanism. The transport and accumulation of various marine-based nanoparticles including chitosan NPs, alginate NPs, fucoidan-coated NPs, liposomes, and marine derived micelles within tumor tissues compared to healthy tissues. Due to the enhanced penetration and retention (EPR) effect and impaired lymphatic drainage in tumors, these NPs preferentially accumulate in the tumor microenvironment. Functionalization with ligands allows for active targeting of cancer cells through surface receptors such as G - protein coupled receptors (GPCRs). After binding, NPs undergo receptor mediated endocytosis, followed by intracellular trafficking to endosomes. Protective surface coatings, such as PEGylation, enhance immune evasion and nuclease resistance, ensuring efficient delivery of therapeutic payloads such as miRNAs or anticancer drugs.

Nanotechnology offers innovative solutions to deliver miRNA-based therapeutics with improved specificity and reduced toxicity. For example, PLGA/PEI nanoparticles have been used to deliver miRNA mimics, resulting in significant tumor suppression in CRC xenograft models (Figure 4) (Yang et al., 2022). Similarly, mesoporous silica nanoparticles loaded with miR-26a have shown efficacy in modulating macrophage activity and reducing tumor growth (Doghish et al., 2025). These nanoparticle-based systems not only improve the stability and bioavailability of miRNA but also enable tissue-specific targeting, overcoming the limitations of naked miRNA-based agents. Despite these advances, challenges such as off-target effects and limited clinical translation remain. Research must continue to optimize delivery systems and validate their efficacy in the clinical setting. Nanotechnology has revolutionized drug delivery systems, particularly for marine-derived compounds, by improving their bioavailability, stability, and targeted delivery.

Marine-derived compounds, such as peptides, alkaloids, and polysaccharides, often face challenges including low solubility, rapid degradation, and poor bioavailability. Nanocarriers, such as liposomes, polymeric nanoparticles, and micelles, offer solutions to these limitations by encapsulating bioactive compounds and protecting them from degradation. Liposomes, spherical vesicles consisting of lipid bilayers, are particularly effective in the delivery of hydrophilic and hydrophobic drugs from the sea. For example, liposomal formulations of marine-derived drugs, such as trabectedin, have demonstrated improved pharmacokinetics and reduced toxicity (Bisht et al., 2025). Polymeric nanoparticles of biodegradable polymers such as polylactic acid (PLA) and poly (lactic-co-glycolic acid) (LPGA) are also commonly used to encapsulate marine drugs and ensure controlled and sustained release. Micelles formed by amphiphilic molecules are another promising nanocarrier system for the delivery of hydrophobic marine drugs, improving their solubility and bioavailability. Recent advances in nanocarrier systems include the development of hybrid nanoparticles that combine the advantages of different nonmaterials. For example, lipid-polymer hybrid nanoparticles have been used to deliver marine-derived anticancer drugs with high loading efficiency and controlled release. These hybrid systems also enable surface functionalization with targeted ligands, improving the specificity of drug delivery to cancer cells.

### 3.5 Comparative insights: marine-derived and conventional nanocarriers

Marine-derived nanocarriers have several advantages when compared with traditional synthetic systems (Table 4). They are naturally biocompatible, and their biodegradability is also a significant benefit. Additionally, they often possess inherent bioactivity, and in the case of poly-specificity, have anti-inflammatory, antimicrobial, or antioxidant properties, which can also work synergistically with a therapeutic payload (Teixeira et al., 2025; Wang et al., 2024). The global shift towards sustainable biomedical solutions is matched by marine polysaccharides (including chitosan, alginate, and carrageenan) and lipids (coming from marine sources) that can offer a source of renewable and environmentally responsible material (Teixeira et al., 2025; Tie and Tan, 2022). Similarly, all polysaccharides and lipids vary in structure, offering numerous options for modification that enable targeted delivery and controlled release of drugs (Wang et al., 2024; Tie and Tan, 2022). However, despite the potential benefits, there are also some limitations to using marine-derived materials, therefore suppressing broader applications (Rajendran & Vadakkepushpakath, 2024). Variability across batches in marine biopolymers can pose a challenge to reproducibility and standardization due to variability in source (seaweed has various species and sometimes strains), issues with the season, and how the material has been extracted (Teixeira et al., 2025; Rajendran & Vadakkepushpakath, 2024). They may have lower mechanical stability and loading efficiency than synthetic nanocarriers, requiring further modifications or blending with synthetic polymers to improve efficacy and efficiency (Tie and Tan, 2022). From a translational standpoint, although the preclinical data show promise for marine-derived nanocarriers, moving these into the clinic can be problematic (Darghiasi et al., 2025; Teixeira et al., 2025; Wu et al., 2024).

**TABLE 4:** Comparison between Marine-derived with conventional nanocarriers.

Attribute	Marine-derived nanocarriers	Conventional nanocarriers	References
Biocompatibility	High	Variable, often lower	Chellapandian et al. (2025); Alshawwa et al. (2022)
Biodegradability	High	Depends on material	Chellapandian et al. (2025); Alshawwa et al. (2022)
Sustainable Sourcing	Yes	Often petroleum-based/limited	Teixeira et al. (2025); Alshawwa et al. (2022)
Reproducibility	Challenging	Generally higher	Teixeira et al. (2025)
Stability (storage/use)	Potential issues	More predictable	Teixeira et al. (2025)
Scalability	Limited by source/cultivation	Well-established for many types	Teixeira et al. (2025)
Regulatory Approval	Emerging, challenging	More established pathways	Teixeira et al. (2025)
Long-term Safety Data	Limited, ongoing research	More available for many materials	Teixeira et al. (2025)

For marine-derived systems, recent advances expose regulatory bottlenecks because of their natural product status, including safety issues surrounding contaminants, impurities, and endotoxins; the complexity of purification steps; limited industrial manufacturing scalability; and the implications for GMP (Darghiasi et al., 2025; Teixeira et al., 2025; Wu et al., 2024). Limited scalability and GMP manufacturing are also issues, and as such, cost-effectiveness and consistency of application would be barriers to broad implementation (Darghiasi et al., 2025; Wu et al., 2024). Finally, our understanding of long-term toxicity and immunogenicity profiles is lacking, which would delay any clinical approvals. To conclude, marine-derived nanocarriers can offer a sustainable and functional alternative to conventional systems, but we must first address key areas such as standardization, scalability, and regulatory hurdles before translating promising laboratory discoveries into clinically approved therapies (Darghiasi et al., 2025).

### 3.6 Marine-inspired nanoparticles for targeted therapy

Marine-derived compounds play a pivotal role in the creation of nanoparticles that exhibit distinctive properties for targeted drug delivery (Jeong et al., 2022). One prominent example is chitosan, a polysaccharide derived from marine crustaceans, which has gained popularity in nanoparticle development due to its biocompatibility, biodegradability, and mucoadhesive properties. Chitosan nanoparticles have effectively delivered marine-derived anticancer agents like bryostatin, facilitating improved cellular uptake and targeted delivery to tumor sites (Wu et al., 2022). Another notable instance involves alginate, a polysaccharide obtained from brown algae, which is utilized to create nanogels for drug delivery. These alginate-based nanogels can encapsulate both hydrophilic and hydrophobic drugs, offering controlled release and protection against enzymatic degradation. They have been used to deliver marine-derived substances, such as fucoidan, a sulfated polysaccharide known for its anticancer and anti-inflammatory effects. Additionally, marine-derived proteins and peptides have been used to fabricate nanoparticles with specific targeting abilities. For example, nanoparticles functionalized with marine-derived peptides display enhanced binding affinity for receptors on cancer cells, thereby increasing the efficacy of drug delivery. These developments underscore the promising potential of marine-inspired nanoparticles for the formulation of targeted therapies for various diseases (Chuang et al., 2024). Marine Compounds and nannoformulated Marine Drugs for mi RNA Modulation.

### 3.7 Nanoformulated marine drugs for dual miRNA and immune modulation

Nanoformulated marine drugs offer an innovative approach for the combined modulation of miRNAs and immune signaling pathways in CRC. This section shifts the focus from general discussions on miRNA delivery to the unique dual functionalities of these formulations. For instance, marine-derived compounds, such as spongistatin, have been encapsulated in nanoparticles, enabling the delivery of miRNA mimics while simultaneously activating immune pathways, such as cGAS-STING (Saeed et al., 2021). Preclinical models have demonstrated the effectiveness of these formulations in boosting the immune response against CRC tumors while silencing the oncogenic miRNAs. Notably, nanoparticles loaded with miR-16 mimics and spongistatin have been shown to inhibit the NF-κB pathway, leading to reduced inflammation and tumor advancement. This dual capability underscores the potential of nanoformulated marine drugs as versatile therapeutic agents.

### 3.8 Microfluidic approaches for nanoparticle synthesis

Microfluidic technology has dramatically improved the production of nanoparticles for marine drug delivery by providing accurate control over their size, shape, and composition. These systems allow for the rapid mixing of lipids and aqueous phases, resulting in uniform lipid nanoparticles with high drug-loading efficiency. For example, lipid nanoparticles synthesized using microfluidic methods have successfully delivered marine anticancer agents, such as trabectedin, promoting enhanced stability and controlled release. This approach is also suitable for the creation of polymeric nanoparticles and nanogels, supporting reproducible and scalable production tailored to meet specific therapeutic requirements (Lopalco et al., 2024).

### 3.9. Safety and toxicity considerations of nanoformulated marine drugs

Marine-based nanoparticles hold significant therapeutic promise; a thorough safety assessment is crucial. Parameters such as particle size, surface charge, and composition impact biocompatibility and potential toxicity. Specific formulations, especially metal-based marine nanoparticles, have been associated with oxidative stress and cytotoxicity at high doses. To reduce these risks, biodegradable materials such as chitosan and alginate are recommended. Extensive *in vitro and in vivo* research is necessary to evaluate the biodistribution, immune response, and long-term safety of these nanoparticles, in accordance with regulatory guidelines (Afzal et al., 2022).

### 3.10 Synergistic effects of marine compounds and nanocarriers in miRNA delivery

The synergistic usage of marine compounds in conjunction with nanocarriers to improve miRNA changes. Marine-derived compounds, like chitosan, are used as nanocarrier materials because of their biocompatibility and efficient binding abilities with miRNAs. Recently, Saeed et al. (2021) reported that chitosan nanoparticles containing miR-34a mimics have proven effect in suppressing tumors in CRC models when they target the Wnt/beta-catenin pathway. The stability and bioavailability of miRNA therapeutics have been improved by incorporating marine-derived lipids into liposomal formulations. These hybrid systems utilize the natural anticancer properties of marine compounds to enhance miRNA delivery and leverage these properties for anticancer applications.

### 3.11 Advances in miRNA delivery systems: beyond nanoparticles

While previous reports have discussed nanoparticle-based delivery systems, some studies focus on alternative delivery methods for miRNA-based therapies (Ganapathy and Ezekiel, 2019; Mehrgou et al., 2020). Viral vectors, such as lentiviruses and adeno-associated viruses (AAVs), have been explored for their ability to deliver miRNA mimics or inhibitors with high efficiency and specificity. For instance, AAV-mediated delivery of miR-34a mimics has demonstrated significant tumor suppression in CRC preclinical models by targeting SIRT1 and E2F3 (Ganapathy and Ezekiel, 2019). Additionally, exosome-based delivery systems have gained attention due to their natural biocompatibility and ability to cross biological barriers (Mehrgou et al., 2020). These systems offer advantages over synthetic nanoparticles, including reduced immunogenicity and enhanced stability; however, scalability and manufacturing remain significant challenges.

## 4. Conclusions and future perspectives

This review underscores the pivotal role of miRNAs in the development of CRC, emphasizing their dual roles as both tumor suppressors and oncogenes. Tumor-suppressive miRNAs, including miR-145, miR-148a, and the miR-200 family, hinder CRC progression by targeting oncogenic pathways such as mTOR, TGFβ/SMAD, and EMT. In contrast, oncogenic miRNAs like miR-21 and miR-17 facilitate tumor growth by inhibiting tumor suppressor genes such as PTEN and RND3. The dysregulation of miRNAs is closely associated with various processes, including metastasis, chemoresistance, and interactions with the gut microbiome, highlighting their potential as both diagnostic biomarkers and therapeutic targets. Compounds such as fucoidan, astaxanthin, and chlorogenic acid, have shown promise in modulating miRNA expression. These compounds not only influence miRNA-related pathways but also possess antioxidant, anti-inflammatory, and anti-metastatic properties, positioning them as excellent candidates for integrative therapies.

The convergence of nanotechnology and miRNA-based therapeutics has propelled the field forward, facilitating targeted delivery and improved stability of miRNA mimics and inhibitors. Nano-formulated marine drugs, including chitosan nanoparticles and liposomal formulations, have shown considerable effectiveness in preclinical models of CRC by merging miRNA modulation with the natural anticancer properties of marine substances. However, obstacles such as off-target effects, challenges in clinical translation, and the scalability of delivery systems still pose significant hurdles. Future studies should focus on refining delivery methods, utilizing bioinformatics resources such as miRDB and TargetScan, and investigating combination therapies that integrate miRNA-focused treatments with traditional strategies, including chemotherapy and immunotherapy. By addressing these issues, miRNA-targeted therapies, particularly those incorporating marine-derived compounds, have significant potential to improve CRC treatment outcomes and expand the boundaries of precision oncology.
